# Supplementary material for: Genomic and microenvironmental heterogeneity shaping epithelial-to-mesenchymal trajectories in cancer
Source: Nat Commun. 2023 Feb 11;14:789. doi: 10.1038/s41467-023-36439-7 (PMC9922305; doi:10.1038/s41467-023-36439-7)
Supplement: Supplementary file 1 — Supplementary Information [file 41467_2023_36439_MOESM1_ESM.pdf]

SUPPLEMENTARY INFORMATION

Genomic and microenvironmental heterogeneity shaping epithelial-to-mesenchymal trajectories in cancer

Guidantonio Malagoli Tagliazucchi[1,#], Anna J Wiecek[1,#], Eloise Withnell[1], Maria Secrier[1,*]

[1] UCL Genetics Institute, Department of Genetics, Evolution and Environment, University College London, UK

[#] These authors contributed equally.

*To whom correspondence should be addressed (msecrier@ucl.ac.uk)

SUPPLEMENTARY FIGURES

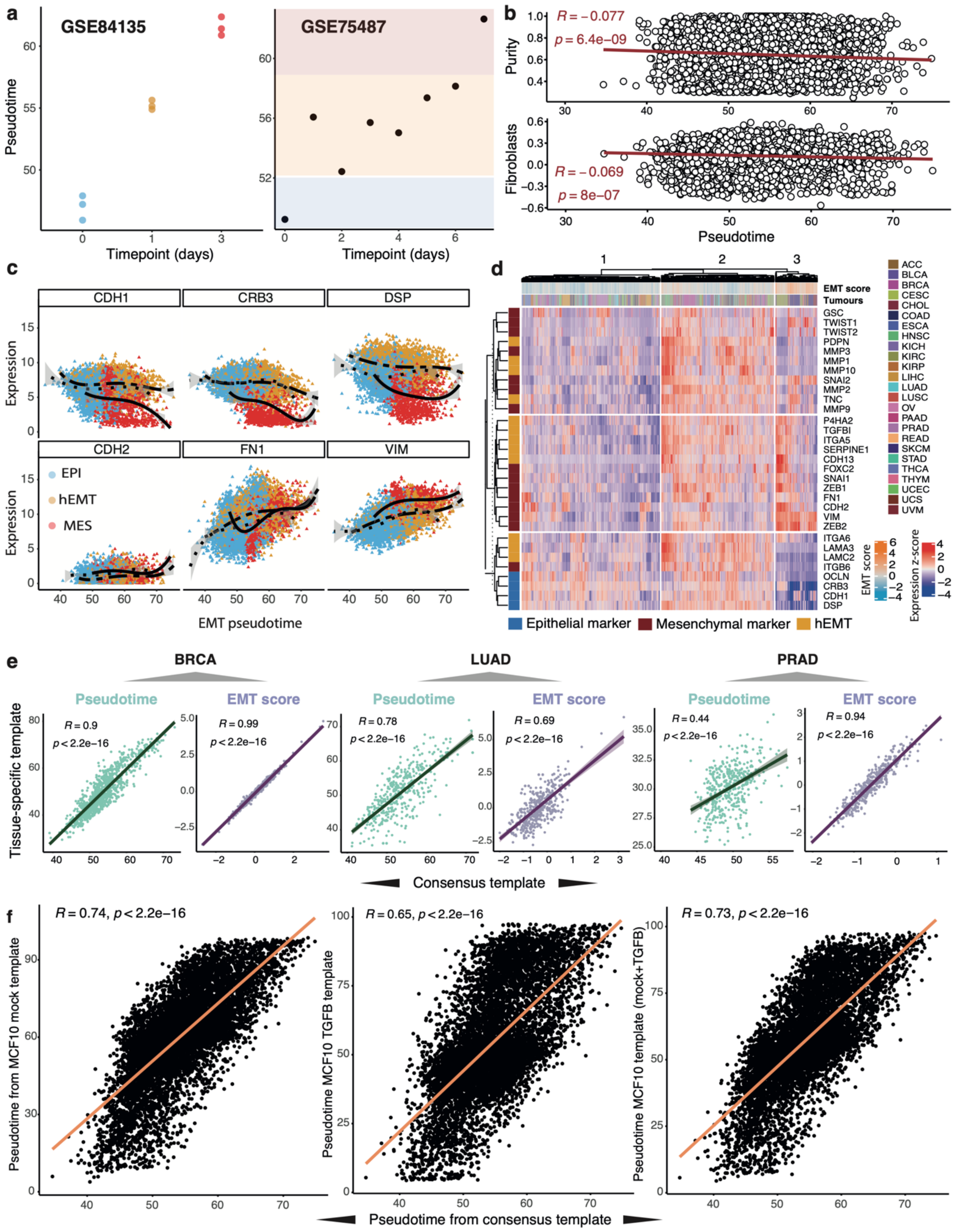

Supplementary Figure 1. Validation of EMT trajectory reconstruction methodology. (a) Application of the EMT trajectory reconstruction method in two longitudinal datasets: GSE84135, a time course EMT transition experiment in hSAEC airway epithelial cells, and GSE75487, 7 day EMT transformation of H358 non-small cell lung cancer cells under doxycycline treatment to induce Zeb1. The pseudotime estimate increases with time as

expected for gradually transforming cells. Distinct colours indicate biological replicates at different time points. (b) The EMT pseudotime estimates for TCGA cancers plotted against tumour purity (Pearson correlation statistic = -0.076, 95% CI [-0.10, -0.05], df = 5,720, p = 6.4e-09) and fibroblast infiltration (Pearson correlation statistic = -0.069, 95% CI [-0.10, -0.04], df = 5,108, p = 8e-07). There is no notable correlation observed with either parameter after correcting for tumour purity. (c) Expression of epithelial/mesenchymal markers along the EMT pseudotime derived from single cell data. Each dot represents a TCGA sample and is coloured according to the assigned EMT state. (d) Heat map highlighting the pan-cancer expression of known EMT markers (coloured by their state-specificity). The heat map colour gradient indicates the expression of selected markers in every TCGA sample profiled. (e) Correlation between pseudotime estimates and EMT scores obtained from the consensus template versus tissue-specific single cell templates, shown for three independent cancer tissues (BRCA – breast cancer; LUAD – lung adenocarcinoma; PRAD – prostate adenocarcinoma). The x axis depicts the estimates calculated with a pan-cancer reference, and the y axis with a tissue-specific reference. Pseudotime and EMT score analyses are shown in different colours. The Pearson correlation coefficients and p-values are displayed in the plots. (f) Correlation between the EMT pseudotime estimates obtained from the consensus template versus the MCF10 template under "mock" (spontaneous) EMT induction, TGFB induction or both. The Pearson correlation coefficients and p-values are displayed in the plots.

Source data are provided as a Source Data file.

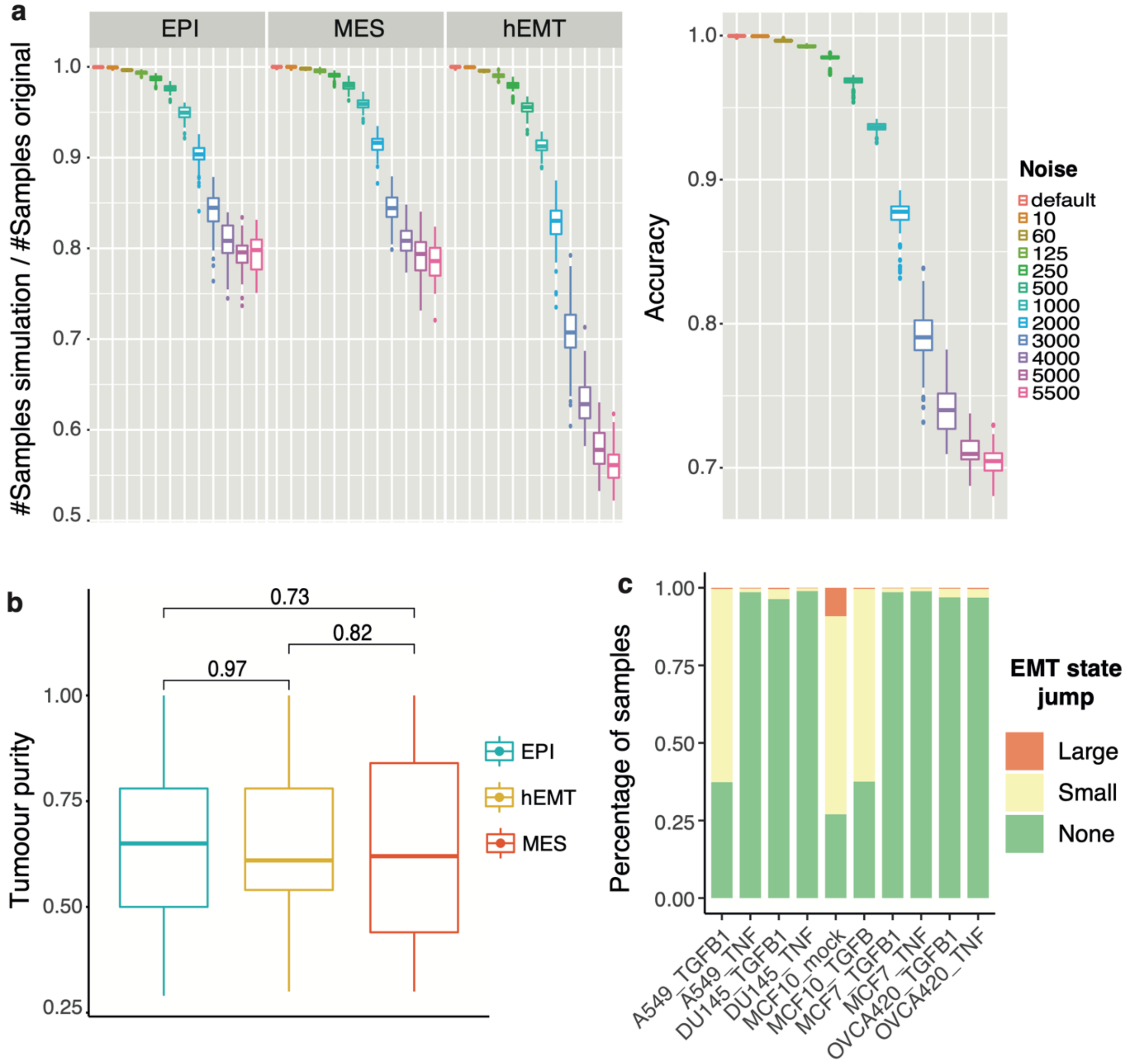

Supplementary Figure 2. Robustness of the HMM-inferred EMT macro-states. (a) Left panel: Fraction of samples correctly assigned to the original epithelial (n=3,388), hEMT (n=2,764) and mesenchymal (n=1,028) HMM state with increasing levels of gene expression noise in the original data (noise levels are indicated by the boxplot colours). Right panel: Accuracy of predicting the original HMM states with increasing levels of gene expression noise in the original data (n=7,180), defined as the fraction of correct assignments. The centerline of boxes depicts the median values; the bottom and top box edges correspond to the first and third quartiles. (b) Tumour purity compared between the three discrete EMT macro-states pan-cancer. (c) Agreement in EMT macro-state assignment when using a consensus pan-cancer template versus individual tissue-specific templates. The plot displays the fraction of samples with identical assignment (no EMT state jump), with minor disagreement in assignment (small EMT state jump, e.g. from EPI to hEMT or from hEMT to MES), and with major disagreement in assignment (large state jump, i.e. from EPI to MES) for each single cell reference. Fraction of samples showing agreement/disagreement of assignment are depicted by different colours.

Source data are provided as a Source Data file.

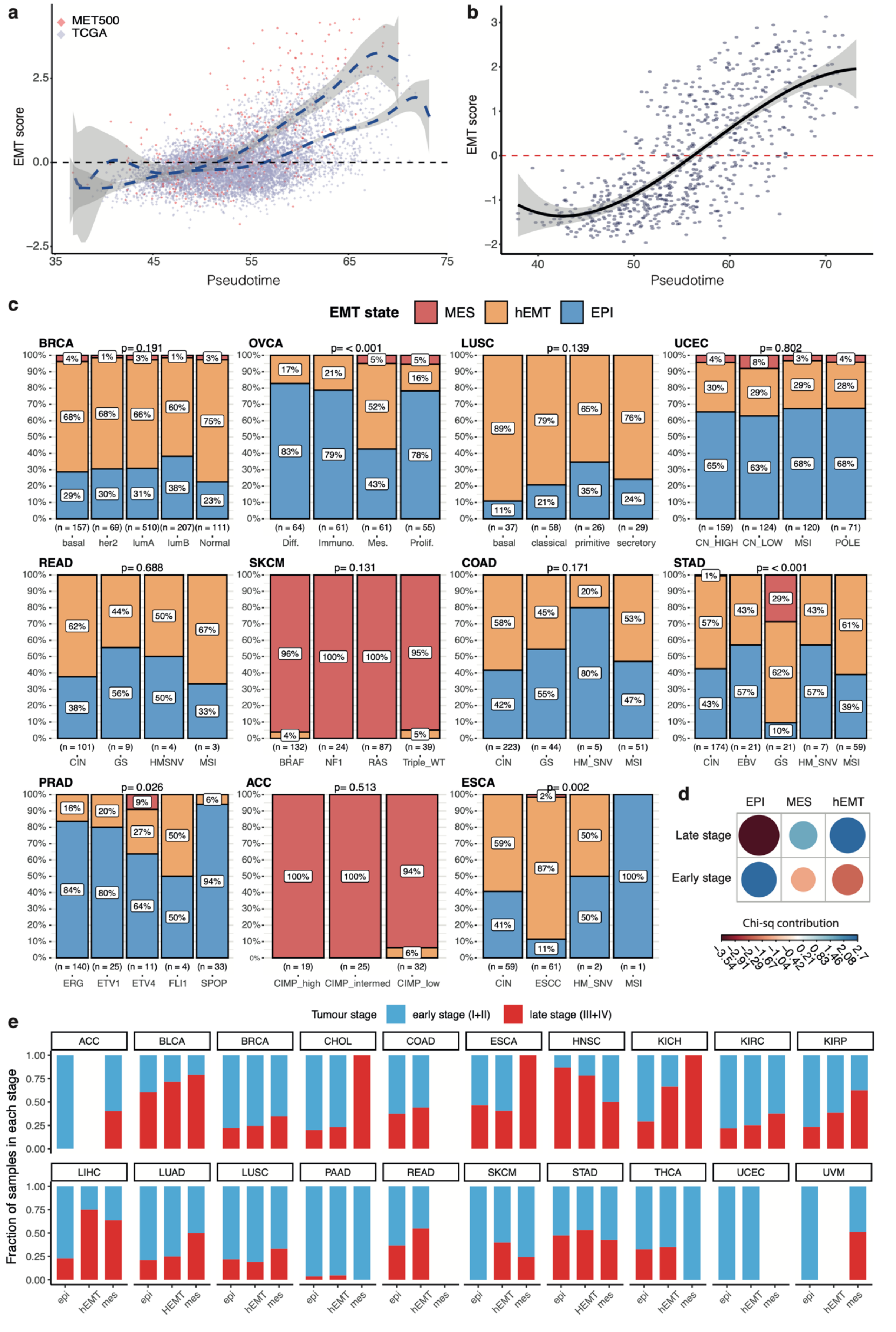

Supplementary Figure 3. Pseudotime reconstruction and EMT macro-state distributions by tissue type and cancer stage. (a) EMT scores of the MET500 and TCGA

samples plotted along the EMT pseudotime derived from single cell data. Every dot corresponds to a sample and it is coloured according to cohort of origin. (b) EMT scores of the CCLE samples plotted along the EMT pseudotime. (c) EMT state distribution by molecular cancer subtypes. The different colours depict the assigned EMT macro-state by our method. (d) Relation between the EMT states and the clinical cancer stage, computed using Chi-square statistics. The circles represent the associations between EMT states and cancer stage (early/late). The colour and size define the strength of the association. (e) Tumour stage distribution across cancer types and EMT macro-states. Early and late stage cancers are depicted in different colours.

Source data are provided as a Source Data file.

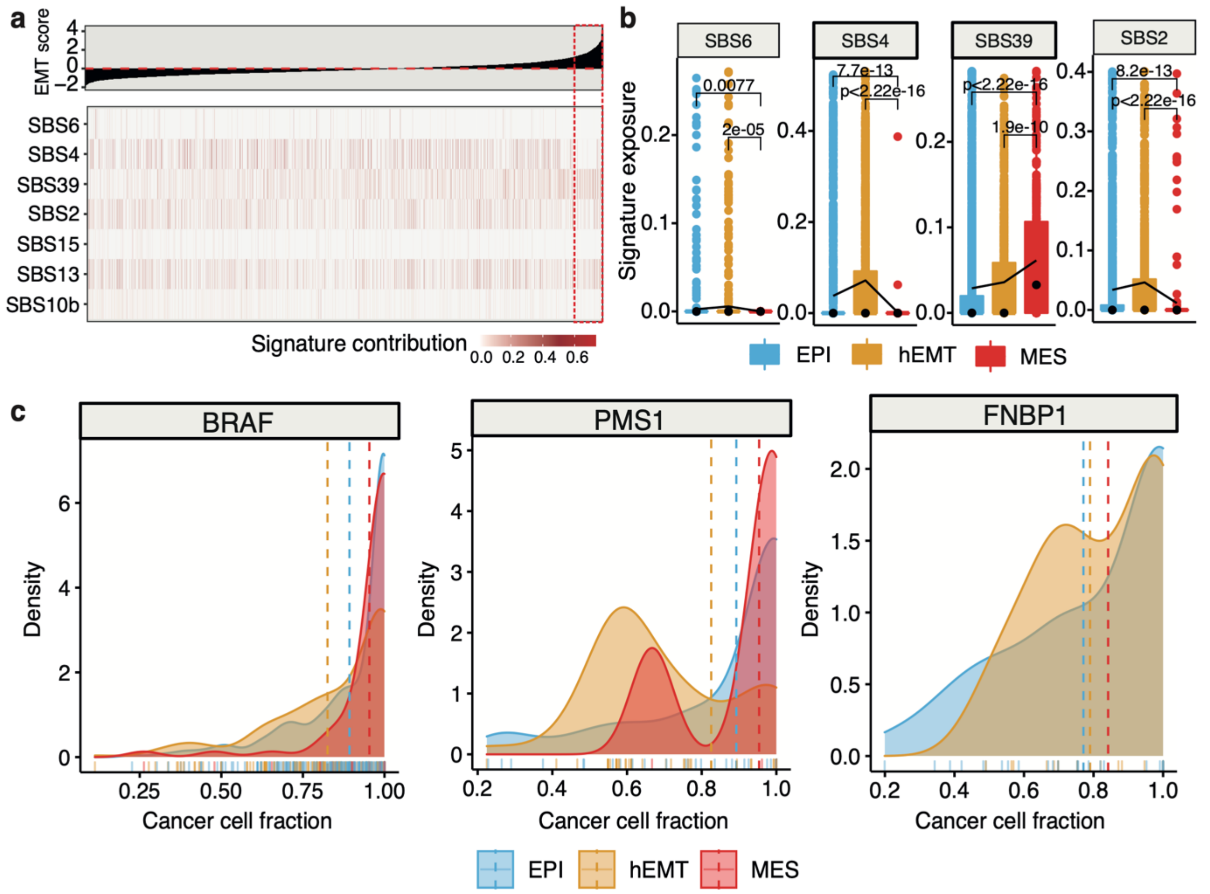

Supplementary Figure 4. Genomic drivers of EMT macro-states. (a) Mutational signature exposures across TCGA samples sorted by EMT score. Only mutational signatures that were significantly linked with EMT from the linear mixed models are displayed. The corresponding EMT scores are displayed above. Every row corresponds to one sample from TCGA for which the mutational signature detection was performed. The colour gradient in the heat map depicts the contribution of individual signatures to every sample, ordered by overall EMT score. (b) Signature contributions from SBS6 (mismatch repair deficiency), SBS4 (smoking), SBS39 (unknown) and SBS2 (APOBEC) compared between the three EMT states, depicted in different colours. The pairwise Wilcoxon rank-sum test p-values are indicated in the plots. The adjoining black line indicates the group means. The box boundaries indicate the first and third quartiles. (c) Cancer cell fraction of genomic markers showing significantly distinct distribution between EMT states. Every density area corresponds to independent biological samples from TCGA belonging to one EMT category, as depicted by the respective colour.

Source data are provided as a Source Data file.

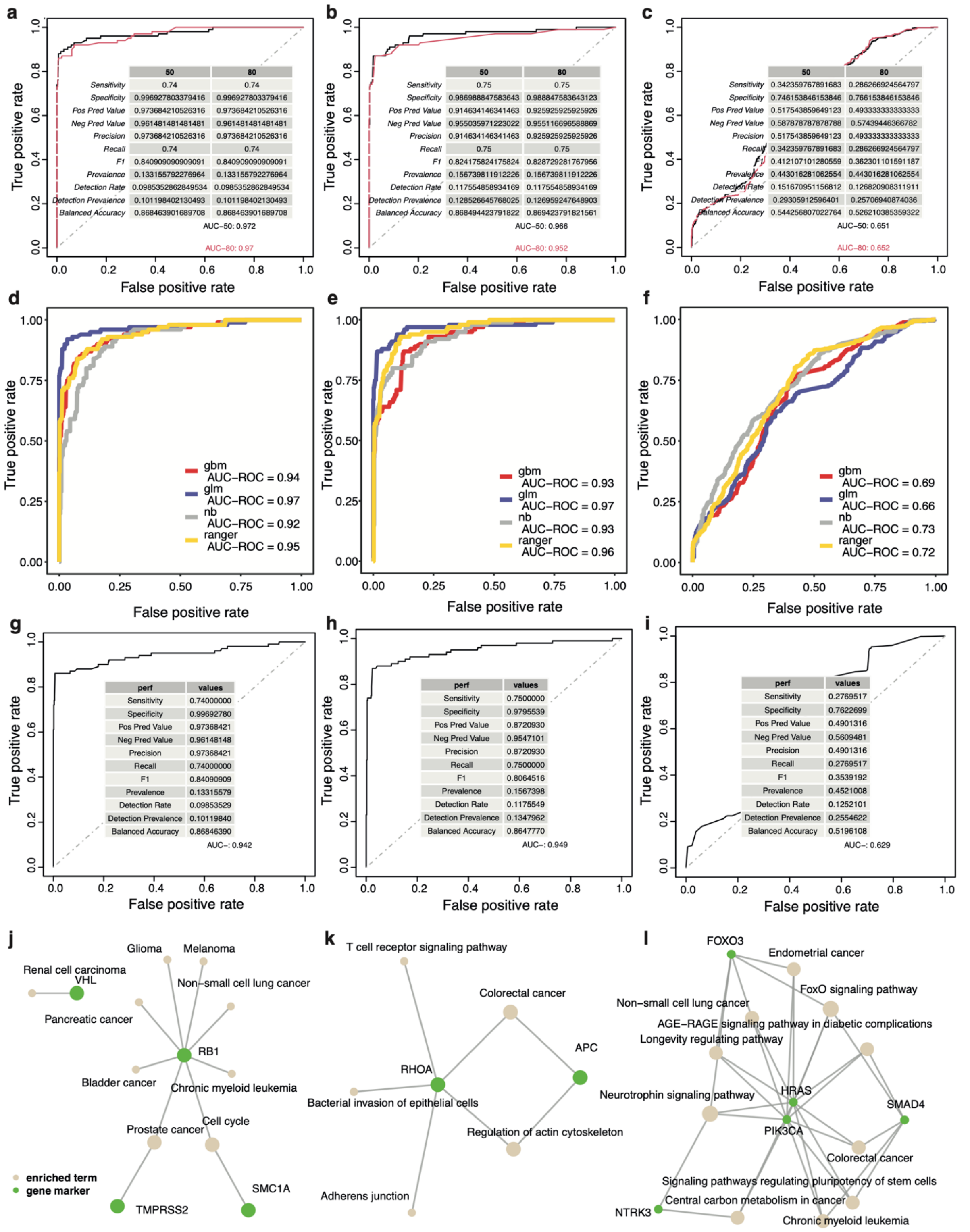

Supplementary Figure 5. Discovery of genomic events linked with the EMT macro-states. (a-c) ROC curves and statistics for the lasso model predictions based on genomic markers distinguishing between MES and EPI (a), MES and hEMT (b), or hEMT and EPI (c) states, respectively. The curves corresponding to the two models retaining 80% and 50% of the features, respectively, are coloured distinctly. (d-f) ROC curves for predictions based on genomic markers obtained with the lasso procedure and tested using different machine learning approaches, for the MES versus EPI (d), MES and hEMT (e), or hEMT and EPI (f)

models, respectively. The curves are coloured to indicate the type of machine learning model employed: gbm – gradient boosting model; glm – generalised linear model; nb – Naïve Bayes; ranger – fast implementation of random forest. (g-h) Same as (a-c) but for random forest based models. (j-l) Pathway enrichment for gene events significantly distinguishing the the MES from the EPI group (j), the MES from the hEMT group (k) and the hEMT from the EPI group (l). The colours of the nodes indicate the genes and associated enriched terms.

Source data are provided as a Source Data file.

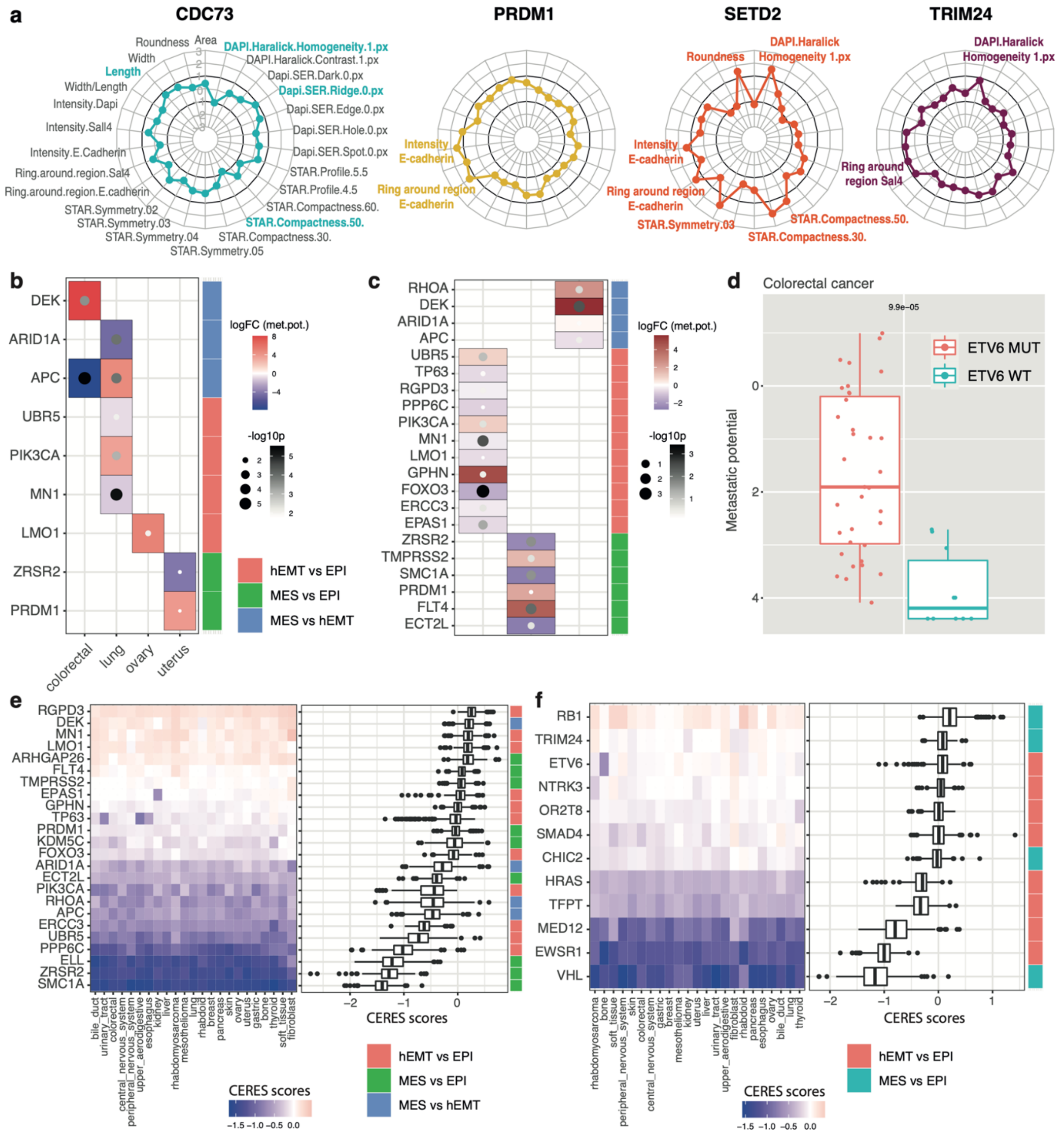

Supplementary Figure 6. Validation of the pan-cancer genomic associations with EMT in external datasets. (a) Gene knockdown effects on various measurements of migration-related cell integrity in mouse embryonic fibroblasts (data from Penalosa-Ruiz et al[1]). The radial plots show the mean z-score depicting the change in cell measurement across multiple knockdown replicates. Z-scores greater than 1 or less than -1 (above and below the corresponding black circles) suggest significant changes. All measurements are listed for the first gene only in grey text. Coloured text indicates significant changes in phenotype for each gene, e.g. knockdown of PRDM1 and SETD2 leads to an increase in E-cadherin expression intensity and area. (b) Fold changes (logFC) in metastatic potential (met.pot.) across cell lines from CCLE originating from different tissues and harbouring copy number alterations in marker genes of EMT, compared to that of cell lines without the respective alteration. The size and the colours of the dots highlight the significance of the association ($p<0.05$). (c) Similar to (b),

but with the analysis performed pan-cancer rather than at tissue level, ($p<0.05$). (d) An increase in metastatic potential is observed in colorectal cancer cell lines harbouring an ETV6 mutation (MUT, n=34) compared to those without the mutation (WT, n=10) (two-sided Wilcoxon rank-sum test p=9.9e-05). The centerline of boxes depicts the median values; the bottom and top box edges correspond to the first and third quartiles. The whiskers ends indicate the minima and maxima. (e) CERES essentiality scores from DepMap in individual cell lineages for genes harbouring copy number alterations linked with EMT. Negative values indicate increased essentiality. The colours in the heat map indicate the average CERES scores in the cell lines of a specific lineage that harbour the alteration. The boxplots on the right indicate the CERES score distribution across all lineages. The centerline of boxes depicts the median values; the bottom and top box edges correspond to the first and third quartiles. (f) Similar to (e) but considering genes harbouring point mutations.

Source data are provided as a Source Data file.

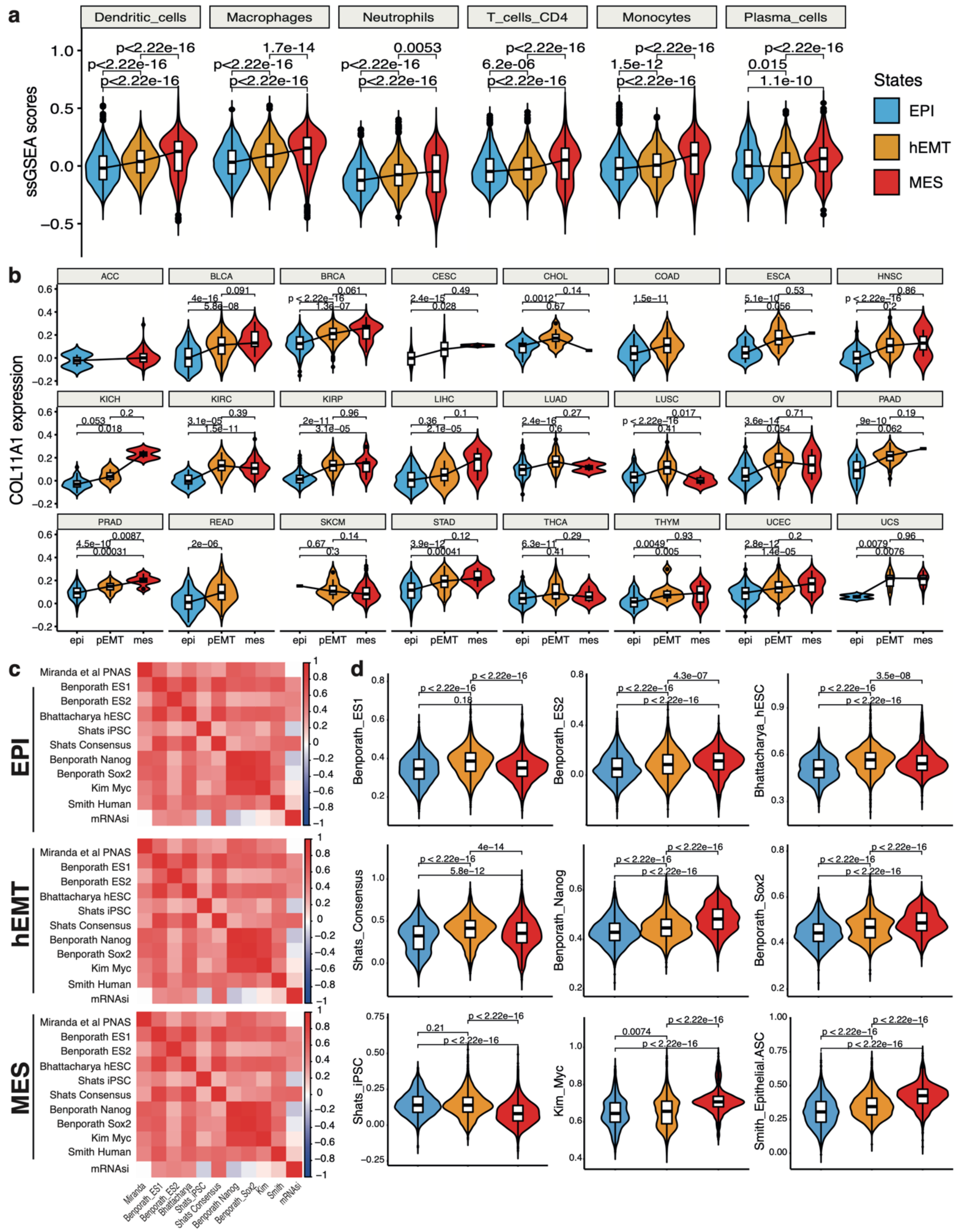

Supplementary Figure 7. Tumour extrinsic properties in relation to EMT. (a) Tumour microenvironment composition compared across biologically independent samples in the epithelial (n=3,388), hEMT (n=2,764) and mesenchymal (n=1,028) states. The groups compared are coloured according to the samples' assigned EMT state. The centerline of boxes depicts the median values; the bottom and top box edges correspond to the first and third quartiles. Two-sided Wilcoxon rank-sum test p-values are displayed. (b) Active fibroblasts infiltration levels (quantified based on COL11A1 expression) compared across biologically

independent samples in the epithelial (n=3,388), hEMT (n=2,764) and mesenchymal (n=1,028) states, by cancer type. The groups compared are coloured according to the samples' assigned EMT state, as in (a). The centerline of boxes depicts the median values; the bottom and top box edges correspond to the first and third quartiles. Two-sided Wilcoxon rank-sum test p-values are displayed. (c) Correlation heat maps of the stemness scores assessed in this study. The strength of the Pearson correlation is highlighted by the colour gradient. (d) Stemness scores compared across biologically independent samples in the epithelial (n=3,388), hEMT (n=2,764) and mesenchymal (n=1,028) states. The groups compared are coloured according to the samples' assigned EMT state, as in (a). The centerline of boxes depicts the median values; the bottom and top box edges correspond to the first and third quartiles. Two-sided Wilcoxon rank-sum test p-values are displayed.

Source data are provided as a Source Data file.

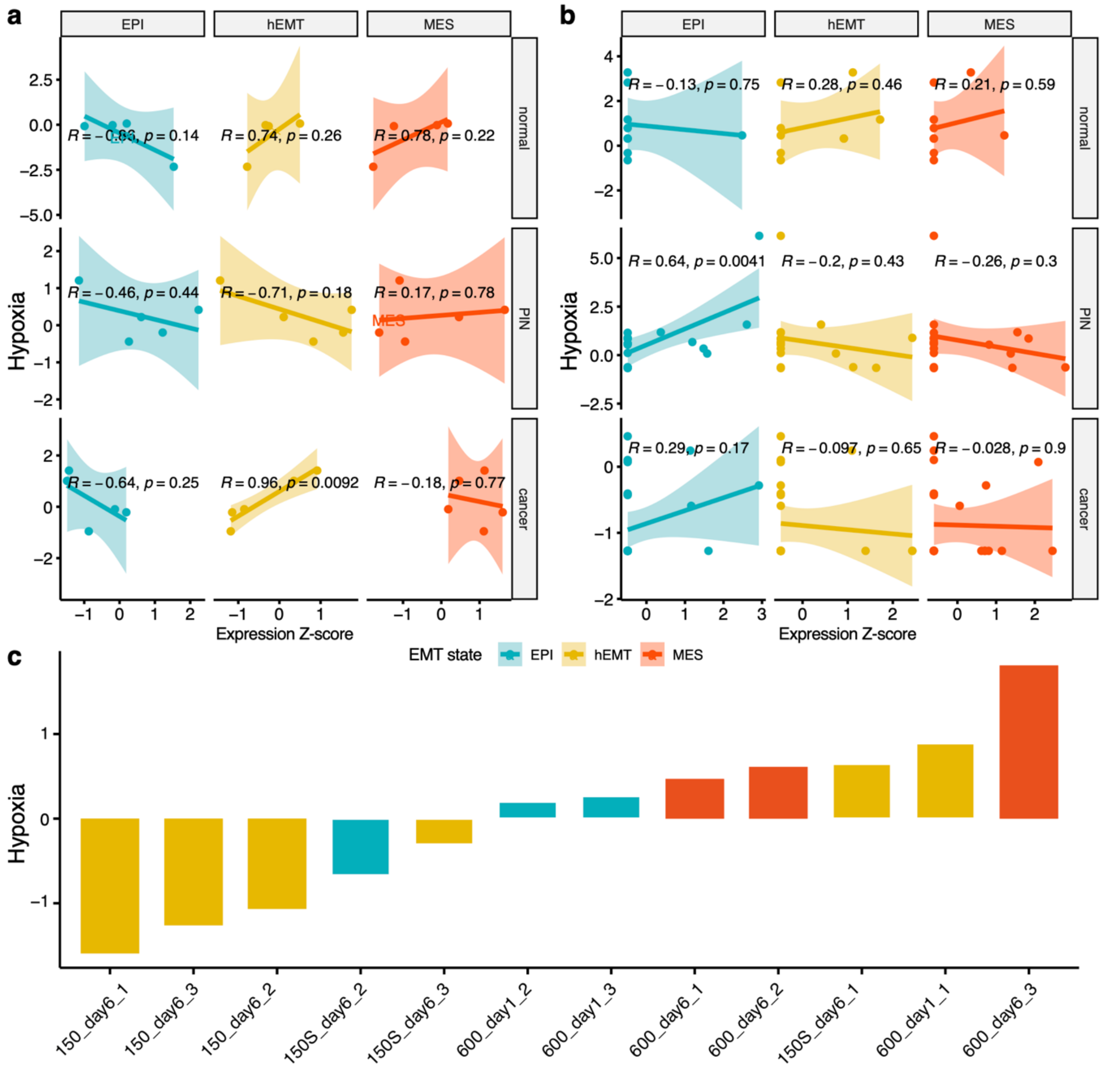

Supplementary Figure 8. Relation between hypoxia and EMT transformation in external datasets. (a) Correlations between hypoxia and EPI, hEMT and MES enrichment scores, respectively, measured in spatially profiled normal, intraepithelial neoplasia (PIN) and prostate cancer samples from tissue section 1.2 from Berglund et al[2]. (b) Correlations between hypoxia and EPI, hEMT and MES enrichment scores, respectively, measured in spatially profiled normal, PIN and prostate cancer samples from tissue section 3.3 from Berglund et al[2]. (c) Hypoxia scores measured in a 3D microtumour model of breast cancer at different days after inducing cellular migration (indicated by the x axis labels). The numbers in the x axis labels indicate biological replicates. Bars are coloured according to the assigned EMT macro-state for the respective sample (like in a), split by quartiles of expression. Source data are provided as a Source Data file.

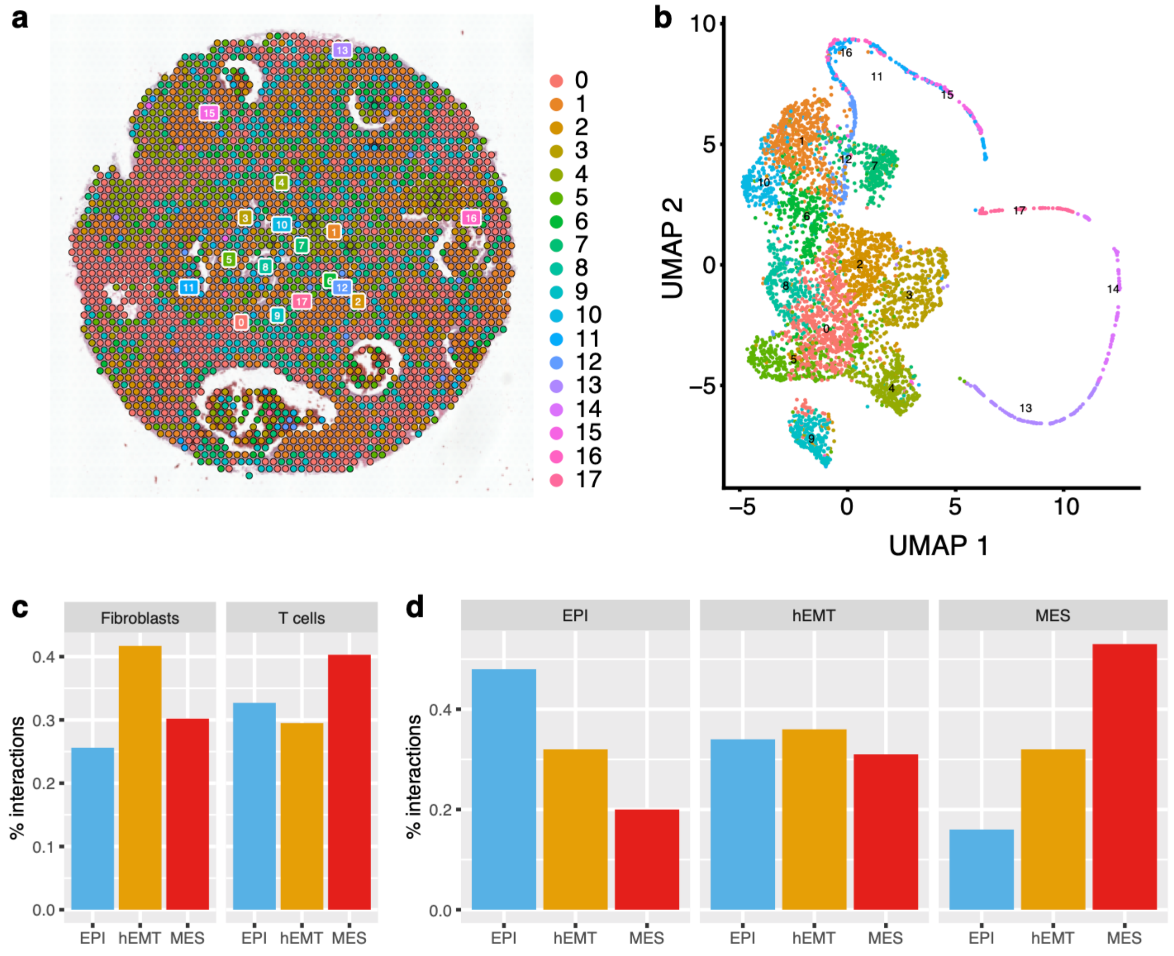

Supplementary Figure 9. Spatial patterns of EMT. (a) Clusters of homogeneous expression profiles annotated within the spatially defined transcriptomic spots are shown for Patient 1. Every cluster is depicted by a distinct colour, and every spot is coloured according to the cluster it was assigned to. (b) Expression clusters visualised using UMAP dimensionality reduction. Every dot corresponds to a spot from the spatial transcriptomics slide and is coloured according to the cluster it was assigned to. (c) Fraction of interactions established between cells in the three EMT macro-states and fibroblasts or T cells in the ST2K dataset. (d) Fraction of interactions established among cancer cells in different EMT macro-states in the ST2K dataset.

Source data are provided as a Source Data file.

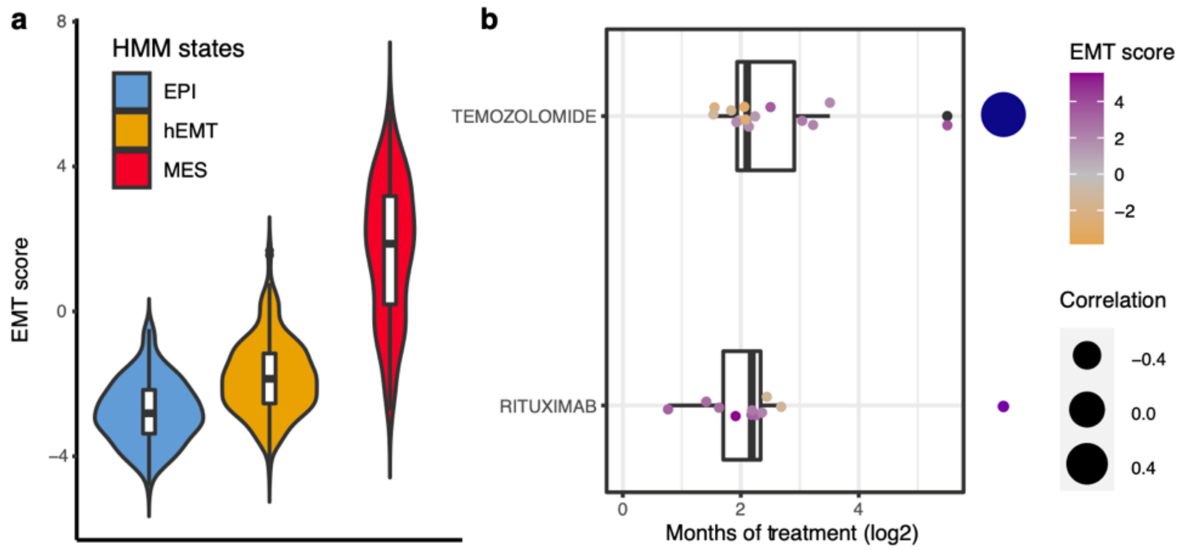

Supplementary Figure 10. Therapeutic relevance of the EMT states in the POG570 cohort. (a) EMT scores are compared across distinct macro-states following EMT reconstruction of the post-treated samples from POG570 (175 epithelial, 231 hEMT and 164 mesenchymal biologically independent samples). The differences observed confirm the classification (Kruskal-Wallis chi-squared (df=2) = 339.24, p-value < 2.2e-16). The centerline of boxes depicts the median values; the bottom and top box edges correspond to the first and third quartiles. (b) Duration of drug treatment is correlated with an EMT score increase (temozolomide, n=14 biologically independent samples) and decrease (rituximab, n=10 biologically independent samples) for selected drugs. The size of the circle is proportional to the Pearson correlation coefficient. Only significant associations are shown. Every dot in the boxplot corresponds to a cell line and is coloured according to its corresponding EMT score. The centerline of boxes depicts the median values; the bottom and top box edges correspond to the first and third quartiles.

Source data are provided as a Source Data file.

SUPPLEMENTARY TABLE CAPTIONS

Supplementary Table 1. Distribution of the TCGA samples in each EMT state across clinical cancer stages.

	Stage I-II	Stage III-IV	Metastasis
**EPI**	1547	715	13
**hEMT**	1351	882	8
**MES**	359	240	322
**Total number of samples**	3257	1837	343
**Fraction of cohort with annotation (%)**	45%	26%	5%

Supplementary Table 2. **Putative EMT biomarkers and literature evidence for links with EMT.** Genes included in at least 80% of the lasso models are listed, along with the model(s) in which they were included and PubMed IDs (PMID) of publications where they are linked with EMT, cell migration or cancer progression. There was weak or no literature evidence for EMT associations for the genes highlighted in red.

Gene	Comparison 1	Comparison 2	PMID
**CHIC2**	MES vs EPI	MES vs hEMT	N/A
**VHL**	MES vs EPI		20068166
**RB1**	MES vs EPI		21502814
**FLT4**	MES vs EPI		33299122
**ZRSR2**	MES vs EPI		33568749
**ARHGAP26**	MES vs EPI	MES vs hEMT	26146084
**ELL**	MES vs EPI		N/A
**PRDM1**	MES vs EPI		23054396
**TMPRSS2**	MES vs EPI		21747944
**ELK4**	EPI vs MES		28446749
**TFG**	EPI vs MES		21596473
**ERBB2**	EPI vs MES	hEMT vs MES	33046882
**ERBB3**	EPI vs MES		32219702
**DEK**	MES vs hEMT		29228721
**APC**	MES vs hEMT		22704062
**EWSR1**	hEMT vs EPI		34885181
**RGPD3**	hEMT vs EPI		N/A
**EPAS1**	hEMT vs EPI		34729113
**SMAD4**	hEMT vs EPI	EPI vs hEMT	29468299

**GPHN**	hEMT vs EPI		N/A
**MN1**	hEMT vs EPI		33105486
**UBR5**	hEMT vs EPI		32363114
**FOXO3**	hEMT vs EPI		33882368
**TP63**	hEMT vs EPI		27681615
**TRIM24**	EPI vs hEMT		33336072
**CDKN2A**	EPI vs hEMT		35429970
**NCKIPSD**	EPI vs hEMT		N/A
**RAF1**	EPI vs hEMT		15308585
**TGFBR2**	EPI vs hEMT		23434068
**SUFU**	EPI vs hEMT		33017570
**CTNNB1**	EPI vs hEMT		27928465
**FNBP1**	EPI vs hEMT		34202606

Supplementary Table 3. Stratification of samples according to hypoxia score and CD44 expression.

	**EPI**	**hEMT**	**MES**
**Hypoxia (+) and CD44 (+)**	387 (5%)	1409 (20%)	347 (5%)
**Hypoxia (+) and CD44 (-)**	636 (9%)	544 (8%)	333 (5%)
**Hypoxia (-) and CD44 (+)**	598 (8%)	555 (8%)	175 (2%)
**Hypoxia (-) and CD44 (-)**	1459 (20%)	348 (5%)	151 (2%)

Supplementary Table 4. Cox regression model results on clinical end points. (a) Results of the multivariate Cox proportional hazards regression model for the overall survival (OS). The mean and standard deviation are reported for each covariate along with the hazard ratios (HR) from the univariable and multivariable models. The p-value for the model is highly significant (p<1e-114). (b) Results of the multivariate Cox proportional hazards regression model for the progression free interval (PFI). The mean and standard deviation are reported for each covariate along with the hazard ratios (HR) from the univariable and multivariable models. The p-value for the model is highly significant (p<1e-79).

**a. OS**		**Summary**	**HR (univariable)**	**HR (multivariable)**
**Age**	Mean (SD)	60.8 (13.8)	1.03 (1.03-1.04, p<0.001)	1.03 (1.03-1.04, p<0.001)
**Gender**	Female	2704 (49.7)	-	-
	Male	2733 (50.3)	1.59 (1.44-1.76, p<0.001)	1.37 (1.24-1.52, p<0.001)
**Tumour stages**	late	1836 (33.8)	-	-
	early	3259 (59.9)	0.40 (0.36-0.45, p<0.001)	0.44 (0.39-0.49, p<0.001)
	metastatic	342 (6.3)	0.63 (0.53-0.75, p<0.001)	0.82 (0.66-1.03, p=0.085)
**EMT state**	EPI	2280 (41.9)	-	-
	hEMT	2237 (41.1)	1.41 (1.26-1.58, p<0.001)	1.35 (1.20-1.51, p<0.001)
	MES	920 (16.9)	1.16 (1.01-1.33, p=0.036)	1.00 (0.84-1.19, p=0.957)
**b. PFI**		**Summary**	**HR (univariable)**	**HR (multivariable)**
**Age**	Mean (SD)	60.8 (13.8)	1.01 (1.01-1.02, p<0.001)	1.01 (1.01-1.02, p<0.001)
**Gender**	Female	2704 (49.7)	-	-
	Male	2733 (50.3)	1.67 (1.52-1.84, p<0.001)	1.43 (1.30-1.58, p<0.001)
**Tumour stages**	late	1836 (33.8)	-	-
	early	3259 (59.9)	0.42 (0.38-0.46, p<0.001)	0.44 (0.40-0.49, p<0.001)
	metastatic	342 (6.3)	1.15 (0.99-1.33, p=0.065)	1.18 (0.97-1.43, p=0.093)
**EMT state**	EPI	2280 (41.9)	-	-
	hEMT	2237 (41.1)	1.05 (0.94-1.17, p=0.354)	1.00 (0.90-1.12, p=0.974)
	MES	920 (16.9)	1.48 (1.31-1.67, p<0.001)	1.04 (0.89-1.22, p=0.622)

Supplementary Table 5. Mutation events impacting overall survival in TCGA. Results of univariate Cox proportional hazards regression model for overall survival based on mutation status are presented. The first column indicates the EMT comparison model the marker was derived from, the second column reports the name of the gene. Hazard ratios (log10) are reported along with confidence intervals, p-values and outcome (Out: 'positive' indicates better prognosis for patients harbouring the mutation in the respective gene, 'negative' indicates worse prognosis). P-values are adjusted for multiple comparisons.

Comparison	Markers	P.value	HR	HR CI (lower)	HR CI (upper)	P.adjusted	Outcome	Significance
MES vs EPI	ERBB2_CN	3.00E-24	1.8	1.6	2	22.4436975	negative	***
MES vs EPI	FLT4_CN	4.00E-20	1.7	1.5	1.9	18.70774393	negative	***
MES vs EPI	TMPRSS2_CN	1.00E-17	1.6	1.4	1.8	16.52287875	negative	***
MES vs EPI	PRDM1_CN	4.90E-20	1.7	1.5	1.9	18.70774393	negative	***
MES vs hEMT	CHIC2_SNV	0.025	0.8	0.3	2.1	1.602059991	positive	*
hEMT vs EPI	CDKN2A_CN	8.70E-82	2.5	2.2	2.8	79.85636076	negative	***
hEMT vs EPI	8p_del	6.30E-37	1.6	1.4	1.7	35.99653947	negative	***
hEMT vs EPI	3p_del	5.90E-51	1.9	1.7	2.1	49.326058	negative	***
hEMT vs EPI	TGFBR2_CN	4.00E-48	1.8	1.6	2	46.79588002	negative	***
hEMT vs EPI	UBR5_CN	2.20E-45	1.8	1.6	2	44.29855538	negative	***
hEMT vs EPI	RAF1_CN	5.80E-47	1.8	1.6	1.9	45.76111791	negative	***
hEMT vs EPI	NCKIPSD_CN	6.50E-47	1.8	1.6	1.9	45.76111791	negative	***
hEMT vs EPI	Genome doublings	8.20E-49	1.8	1.6	2	47.35918742	negative	***
hEMT vs EPI	GPHN_CN	8.60E-44	1.7	1.5	1.9	42.76447155	negative	***
hEMT vs EPI	EPAS1_CN	4.60E-38	1.6	1.4	1.8	37.0873647	negative	***
hEMT vs EPI	FNBP1_SNV	2.10E-19	0.72	0.43	1.2	18.64975198	positive	***
hEMT vs EPI	FNBP1_SNV	2.10E-19	0.72	0.43	1.2	18.64975198	positive	***

REFERENCES

1 Peñalosa-Ruiz, G. *et al.* WDR5, BRCA1, and BARD1 Co-regulate the DNA Damage Response and Modulate the Mesenchymal-to-Epithelial Transition during Early Reprogramming. *Stem Cell Reports* **12**, 743-756 (2019). https://doi.org:10.1016/j.stemcr.2019.02.006

2 Berglund, E. *et al.* Spatial maps of prostate cancer transcriptomes reveal an unexplored landscape of heterogeneity. *Nat Commun* **9**, 2419 (2018). https://doi.org:10.1038/s41467-018-04724-5
