# Supplementary material for: Genomic and microenvironmental heterogeneity shaping epithelial-to-mesenchymal trajectories in cancer
Source: Nat Commun. 2023 Feb 11;14:789. doi: 10.1038/s41467-023-36439-7 (PMC9922305; doi:10.1038/s41467-023-36439-7)
Supplement: Supplementary file 3 — Description of Additional Supplementary Files [file 41467_2023_36439_MOESM3_ESM.pdf]

Description of Additional Supplementary Data Files

Supplementary Data 1. Pseudotime reconstruction of EMT trajectories in TCGA samples. The results of the pan-cancer reconstruction of epithelial-to-mesenchymal trajectories is reported, along with the HMM state of the sample, and the corresponding EMT macro-state and score.

Supplementary Data 2. **Pan-cancer genomic events linked with EMT states.** The results of the lasso models built to distinguish EMT states based on genomic markers are shown. All genes included in at least 50% of the models are listed. The first column indicates the comparison in which a genomic marker (second column) has been identified. Additional details on the genes are provided. The mean coefficients from the lasso model (Mean contribution). The final column annotates the genes comprised within an altered chromosomal arm.

Supplementary Data 3. **EMT gene candidates employed for validation using siRNA screens.** Genes included in at least 50% of the lasso models are listed, along with coefficient details from the respective models, genomic location and PubMed IDs (PMID) of publications where they are linked with EMT, cell migration or cancer progression.
