# Supplementary material for: Genomic and microenvironmental heterogeneity shaping epithelial-to-mesenchymal trajectories in cancer
Source: Nat Commun. 2023 Feb 11;14:789. doi: 10.1038/s41467-023-36439-7 (PMC9922305; doi:10.1038/s41467-023-36439-7)
Supplement: Supplementary file 7 — Reporting Summary [file 41467_2023_36439_MOESM7_ESM.pdf]

nature portfolio

Corresponding author(s): Maria Secrier

Last updated by author(s): Jan 24, 2023

Reporting Summary

Nature Portfolio wishes to improve the reproducibility of the work that we publish. This form provides structure for consistency and transparency in reporting. For further information on Nature Portfolio policies, see our Editorial Policies and the Editorial Policy Checklist.

Statistics

For all statistical analyses, confirm that the following items are present in the figure legend, table legend, main text, or Methods section.

n/a	Confirmed	
☐	☒	The exact sample size (*n*) for each experimental group/condition, given as a discrete number and unit of measurement
☒	☐	A statement on whether measurements were taken from distinct samples or whether the same sample was measured repeatedly
☐	☒	The statistical test(s) used AND whether they are one- or two-sided<br>*Only common tests should be described solely by name; describe more complex techniques in the Methods section.*
☐	☒	A description of all covariates tested
☐	☒	A description of any assumptions or corrections, such as tests of normality and adjustment for multiple comparisons
☐	☒	A full description of the statistical parameters including central tendency (e.g. means) or other basic estimates (e.g. regression coefficient) AND variation (e.g. standard deviation) or associated estimates of uncertainty (e.g. confidence intervals)
☐	☒	For null hypothesis testing, the test statistic (e.g. *F*, *t*, *r*) with confidence intervals, effect sizes, degrees of freedom and *P* value noted<br>*Give P values as exact values whenever suitable.*
☒	☐	For Bayesian analysis, information on the choice of priors and Markov chain Monte Carlo settings
☒	☐	For hierarchical and complex designs, identification of the appropriate level for tests and full reporting of outcomes
☐	☒	Estimates of effect sizes (e.g. Cohen's *d*, Pearson's *r*), indicating how they were calculated

Our web collection on statistics for biologists contains articles on many of the points above.

Software and code

Policy information about availability of computer code

Data collection	All code for data collection was developed in R (retrieval from public databases using custom libraries). The code developed for this purpose has been deposited at the following repository: https://github.com/secrierlab/EMT<br><br>The following R package has been employed for data retrieval: TCGAbiolinks 2.25.3
Data analysis	All code for data analysis was developed in R and Python v3.7. The code developed for this purpose has been deposited at the following repository: https://github.com/secrierlab/EMT<br><br>The following R packages have been employed for data analysis:<br>caret 6.0-93<br>ConsensusTME 0.0.1.9000<br>diagram 1.6.5<br>depmixS4 1.5-0<br>FNN 1.1.3.1<br>ggplot2 3.4.0<br>ggpubr 0.5.0<br>glmnet 4.1-6<br>GSVA 1.46.0<br>MLeval 0.3<br>MOFA v2<br>nnet 7.3-18

pathfindR 1.6.4
SCTransform 0.3.5
Seurat 4.3.0
Stereoscope v.0.2
STUtility 2022 release
survival 3.4-0
survminer 0.4.9
sva 3.42.0
vcd 1.4-10

The following Python packages have been employed for data analysis:
ScanPy 1.9.1
SquidPy 1.2.3

For manuscripts utilizing custom algorithms or software that are central to the research but not yet described in published literature, software must be made available to editors and reviewers. We strongly encourage code deposition in a community repository (e.g. GitHub). See the Nature Portfolio guidelines for submitting code & software for further information.

Data

Policy information about availability of data

All manuscripts must include a data availability statement. This statement should provide the following information, where applicable:

- Accession codes, unique identifiers, or web links for publicly available datasets
- A description of any restrictions on data availability
- For clinical datasets or third party data, please ensure that the statement adheres to our policy

The results published here are based upon publicly available data generated by the TCGA Research Network (https://www.cancer.gov/tcga), MET500 (https://met500.path.med.umich.edu/), MetMap (https://depmap.org/metmap/), GDSC (https://www.cancerrxgene.org/), POG570 (https://www.bcgsc.ca/downloads/POG570/), CCLE (https://sites.broadinstitute.org/ccle/) and GDSC (https://www.cancerrxgene.org/). The following expression datasets from the Gene Expression Omnibus (GEO) have also been employed: GSE17708 [https://www.ncbi.nlm.nih.gov/geo/query/acc.cgi?acc=GSE17708], GSE84135 [https://www.ncbi.nlm.nih.gov/geo/query/acc.cgi?acc=GSE84135], GSE75487 [https://www.ncbi.nlm.nih.gov/geo/query/acc.cgi?acc=GSE75487], GSE16621153 [https://www.ncbi.nlm.nih.gov/geo/query/acc.cgi?acc=GSE166211], GSE75688 accession code [https://www.ncbi.nlm.nih.gov/geo/query/acc.cgi?acc=GSE75688]. The single cell data from Qian et al58 were obtained from https://lambrechtslab.sites.vib.be/en/pan-cancer-blueprint-tumour-microenvironment-0. The spatial transcriptomics data employed in the study were downloaded from https://support.10xgenomics.com/spatial-gene-expression/datasets (10x Genomics Visium slides) and from https://github.com/almaan/her2st (ST2K slides). All data comply with ethical regulations, with approval and informed consent for collection and sharing already obtained by the relevant consortia.
Source data are provided with this paper.

Human research participants

Policy information about studies involving human research participants and Sex and Gender in Research.

Reporting on sex and gender	Sex and gender have not been considered in the design of this study, because it was not considered a key parameter that might determine EMT in cancer. All results presented come from amalgamating human data from both sexes. No filtering of the human data was performed based on sex or gender, and no sex and gender information has been collected for this study.
Population characteristics	The data used in this study is publicly available from the Cancer Genome Atlas. Detailed population characteristics are available publicly at: https://www.cancer.gov/about-nci/organization/ccg/research/structural-genomics/tcga
Recruitment	The data has been collected by TCGA and details on the procedure are available publicly at: https://www.cancer.gov/about-nci/organization/ccg/research/structural-genomics/tcga
Ethics oversight	All data employed in this study are publicly available and ethical consent has already been obtained accordingly. Please see here for more details: https://www.cancer.gov/about-nci/organization/ccg/research/structural-genomics/tcga/history/policies

Note that full information on the approval of the study protocol must also be provided in the manuscript.

Field-specific reporting

Please select the one below that is the best fit for your research. If you are not sure, read the appropriate sections before making your selection.

☒ Life sciences ☐ Behavioural & social sciences ☐ Ecological, evolutionary & environmental sciences

For a reference copy of the document with all sections, see nature.com/documents/nr-reporting-summary-flat.pdf

Life sciences study design

All studies must disclose on these points even when the disclosure is negative.

Sample size	The data used in this study are public (e.g. TCGA, MET500), therefore no initial estimation of the sample size has been performed in this study. The TCGA dataset (7,180 samples) was used as a discovery dataset to describe EMT phenotypes and linked genomic dependencies. The study comprises >7,000 samples, so it is well powered to uncover statistically significant associations when split into three groups. When we applied machine learning methodologies to identify the biomarkers of EMT, the dataset has been split in training set (80%) and test set (20%) to generate the models. Approaches of cross-validation have been also employed to assess the quality of the models. A similar strategy of splitting has been applied also for other analyses (e.g. multinomial logistic regression).<br><br>The following datasets have been used for validation or further confirmation of the findings: MET500 (500 metastatic samples), MetMap (476 cell lines), POG570 (n=570), CCLE cell lines (n=947).<br><br>We chose TCGA to be the discovery dataset as it is the largest publicly available resource with matched genomic and transcriptomic cancer data from various tissues which would allow a pan-cancer analyses. For validation, we selected all datasets that were publicly available and that contained suitable matched genomic (at least whole-exome sequencing) and RNA-seq data. We also analysed EMT phenotypes in cell lines, due to the broad availability of drug response data.<br><br>The Qian et al single cell dataset (n=233,591) was chosen to illustrate the broad applicably of our EMT methodology to single cell data as it comprised four distinct cancer types and a good number of cells sequenced for each sample. The Chung et al (n=515) dataset was also analysed because it contained matched bulk and single cell RNA-seq data, which allowed us to confirm our method captures similar features in bulk as in single cells.<br><br>The breast cancer 10x Visium slides (n=3) and ST2K data (n=36) were chosen to illustrate the applicability of our method to spatial transcriptomics due to their public availability in a field were data are still quite scarce, and because EMT has been well characterised in breast cancer.
Data exclusions	All TCGA cancers that do not have an epithelial origin have been excluded from the analysis, as evaluating an epithelial to mesenchymal transition in non-epithelial cells of origin is less likely to be physiologically relevant. Excluding non-epithelial cancers was a pre-established criterion.
Replication	The analytical procedures implemented here are highly reproducible, consisting of fixed steps and parameters, and the code has been made freely available at the following repository: https://github.com/secrierlab/EMT.<br>We also employed multiple machine learning procedures to ensure the robustness of the analysis. For example, in the case of the selection of the biomarkers associated with EMT, we used a stability selection approach, whereby we repeated the procedure of selection of the variables obtained with several algorithms (lasso, random forest) 1000 times and selected the most frequent biomarkers identified at the end of the iterations.
Randomization	Samples were split into groups according to their EMT profiles as detailed in the methods. The analysis did not involve any random allocation of samples.
Blinding	Blinding is not relevant to this study as it does not involve a clinical trial or treatment allocation.

Reporting for specific materials, systems and methods

We require information from authors about some types of materials, experimental systems and methods used in many studies. Here, indicate whether each material, system or method listed is relevant to your study. If you are not sure if a list item applies to your research, read the appropriate section before selecting a response.

Materials & experimental systems

n/a	Involved in the study	
☒	☐	Antibodies
☒	☐	Eukaryotic cell lines
☒	☐	Palaeontology and archaeology
☒	☐	Animals and other organisms
☒	☐	Clinical data
☒	☐	Dual use research of concern

Methods

n/a	Involved in the study	
☒	☐	ChIP-seq
☒	☐	Flow cytometry
☒	☐	MRI-based neuroimaging
